# In vitro ballooned hepatocytes can be produced by primary human hepatocytes and hepatic stellate cell sheets

**DOI:** 10.1038/s41598-022-09428-x

**Published:** 2022-03-29

**Authors:** Nobuhiro Hasui, Katsuhisa Sakaguchi, Tetsuya Ogawa, Yoshihiro Sakamoto, Tatsuya Shimizu

**Affiliations:** 1 Institute of Advanced Biomedical Engineering and Science, TWIns, Tokyo Women’s Medical University, 8-1 TWIns Kawada-cho, Shinjuku, Tokyo 162-8666 Japan; 2 Department of Gastrointestinal and General Surgery, Kyorin University School of Medicine, Tokyo, Japan; 3 Department of Integrative Bioscience and Biomedical Engineering, Graduate School of Advanced Science and Engineering, TWIns, Waseda University, 2-2 TWIns Wakamatsu-cho, Shinjuku, Tokyo 162-0056 Japan; 4 Ogino Memorial Laboratory, Nihon Kohden Co., Ltd., Tokyo, Japan; 5 Department of Hepato-Biliary-Pancreatic Surgery, Kyorin University Hospital, Tokyo, Japan

**Keywords:** Biomedical engineering, Hepatology, Disease model

## Abstract

Despite the increasing prevalence of Nonalcoholic steatohepatitis (NASH) worldwide, there is no effective treatment available for this disease. “Ballooned hepatocyte” is a characteristic finding in NASH and is correlated with disease prognosis, but their mechanisms of action are poorly understood; furthermore, neither animal nor in vitro models of NASH have been able to adequately represent ballooned hepatocytes. Herein, we engineered cell sheets to develop a new in vitro model of ballooned hepatocytes. Primary human hepatocytes (PHH) and Hepatic stellate cells (HSC) were co-cultured to produce cell sheets, which were cultured in glucose and lipid containing medium, following which histological and functional analyses were performed. Histological findings showed hepatocyte ballooning, accumulation of fat droplets, abnormal cytokeratin arrangement, and the presence of Mallory–Denk bodies and abnormal organelles. These findings are similar to those of ballooned hepatocytes in human NASH. Functional analysis showed elevated levels of TGFβ-1, SHH, and p62, but not TNF-α, IL-8. Exposure of PHH/HSC sheets to a glucolipotoxicity environment induces ballooned hepatocyte without inflammation. Moreover, fibrosis is an important mechanism underlying ballooned hepatocytes and could be the basis for the development of a new in vitro NASH model with ballooned hepatocytes.

## Introduction

Nonalcoholic fatty liver disease (NAFLD) is a metabolic syndrome of the liver and is a general term for diseases in which excessive triglycerides are deposited in hepatocytes. It is estimated that in 10% of patients, NAFLD progresses to irreversible NASH characterized by inflammation and fibrosis, which may eventually lead to liver cirrhosis and cancer^1^. It has been proposed that the pathogenesis and progression of NAFLD/NASH not only is dependent on the genetic background but is also influenced by various environmental factors such as cellular stress due to excessive lipid intake, increased insulin resistance, chronic inflammation of the adipose tissue owing to obesity, and abnormal secretion of adipocytokines^2^. The prevalence of NAFLD is over 25% worldwide and is increasing with an increase in the obese population^1^. At present, there is no effective treatment for NASH; a high unmet medical need. Therefore, the development of novel drugs for treating this disease is urgently needed.

Rodent models of NASH have been developed and studied since the 1990s when the disease concept was framed. However, no animal model completely reflects the pathogenesis of NASH as in humans due to differences in genetic background and nutrient requirements^3^. The histological features of NASH include “ballooned hepatocytes” (BHs), fat accumulation in the liver, hepatic fibrosis, inflammatory cell infiltration, and hepatocyte necrosis. In particular, human BHs are characterized by rounding and enlargement of the entire cell and thinning of the cytoplasm (“ballooning”) and eosinophilic body in hematoxylin–eosin (HE) staining. These features are specific to humans, unlike mouse BHs, which are enlarged mainly due to an increase in the number of accumulated microvesicles^4^. BHs in human NASH are characterized by ballooning and the accumulation of fat droplets in hepatocytes, abnormal cytokeratin (CK) arrangement, abnormal organelles, and Mallory–Denk-body (MDB) formation^5^.

Recently, in vitro models of NASH have been developed using tissue engineering techniques such as spheroid and perfusion culture of human liver-derived cells to circumvent the problems associated with animal models^6^. Whereas several in vitro models show morphological features that are specific to human NASH, such as fat accumulation and fibrosis^7–9^, none of them have been able to generate BHs. In humans, the number of BHs correlates with the severity of NASH, and its semi-quantification is reflected in the NAFLD activity score. Since BHs are potentially a therapeutic target^10^, it is important to develop a model that could produce BHs in vitro. Although BHs are clinically important in the development and progression of NASH, the mechanisms underlying BHs formation are poorly understood.

We previously demonstrated that in vitro cultured primary human hepatocyte (PHH) sheets can maintain liver functions for a long time^11^. We also found that exposure to high glucose and insulin levels induces ballooning in PHHs co-cultured with normal human dermal fibroblast (NHDF)^12,13^.

Herein, we attempted to produce BHs not only with ballooning but also with other features, such as fat accumulation and the presence of Mallory-Denk bodies (MDBs), by fabricating cell sheets with PHH and Hepatic stellate cell (HSC), which are originally involved in liver fibrosis. Moreover, we also tried to uncover the mechanism underlying BH formation and the molecules involved.

## Materials and method

### Hepatic stellate cell culture

HSCs (#HMFHSC, Lot: HMFHSC8275) were purchased from Thermo Fisher Scientific (Waltham, MA, USA). HSCs (passage 1) were seeded at a density of 5 × 10^4^ cells/cm^2^ on collagen-coated dishes (#4020-010, IWAKI, Tokyo, Japan) and maintained in Stellate Cell Medium (#5301, ScienCell Research Laboratories, San Diego, CA, USA) in an incubator with 5% CO_2_ at 37 °C.

### Fabrication of PHH/HSC sheets

**Figure 1:**
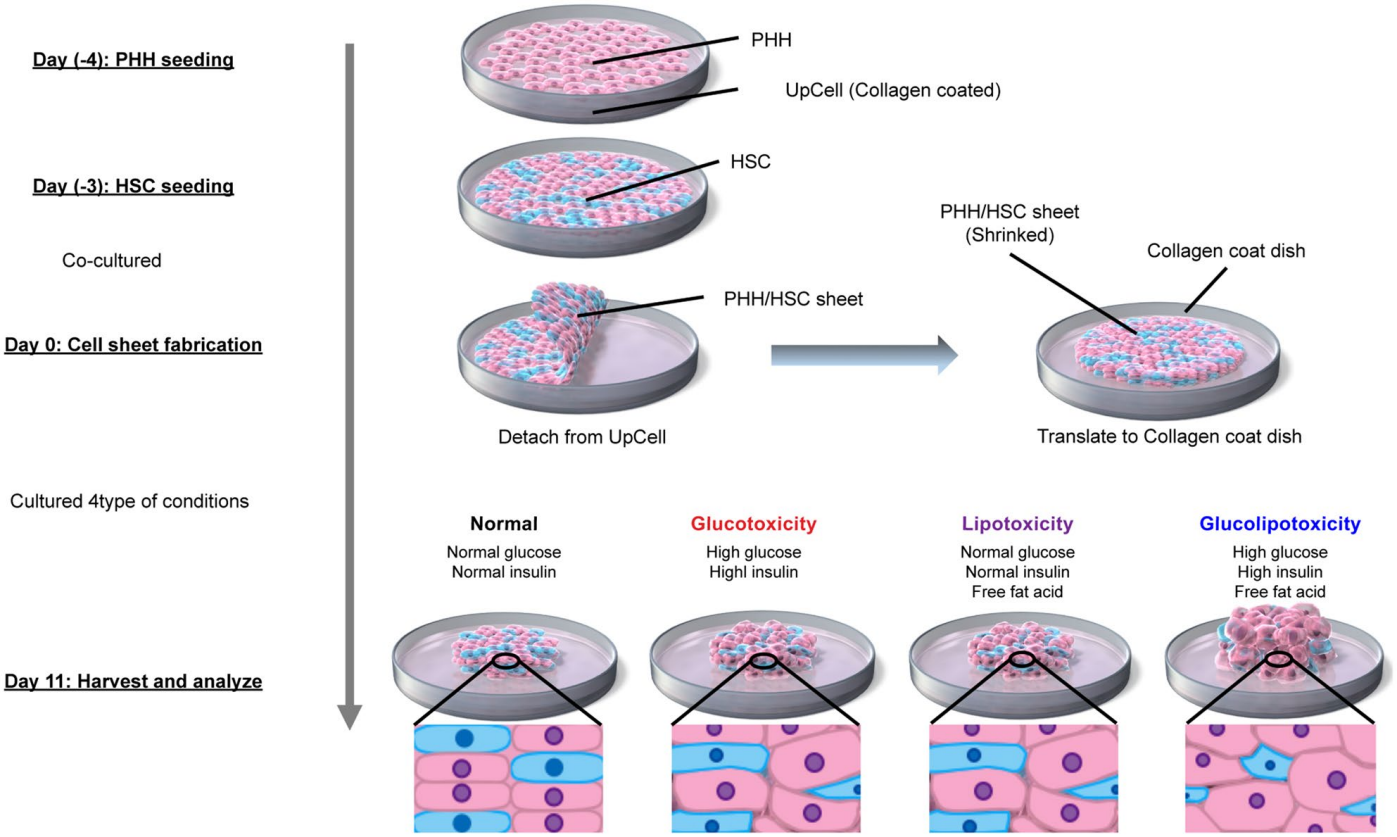
Experiment protocol and images. *PHH* Primary human hepatocyte, *HSC* Hepatic stellate cells.

The protocol is illustrated in Fig. 1. PHHs (#HMSPTS, Lot: Hu8200, Hu8317, Hu 1652) were purchased from Thermo Fisher Scientific. Temperature-responsive culture dishes (UpCell, #CS3007, #CS3002) were purchased from CellSeed (Tokyo, Japan). On the day of PHH seeding (day 4), the dishes were coated with collagen (70 μg/cm^2^; #354236, Corning, New York, NY, USA) and iMatrix (0.25 μg/cm^2^; #892011, Takara Bio, Shiga, Japan) and incubated at 37 °C for 5 h. The coated dishes were washed twice with PBS at room temperature. PHHs were thawed in Cryopreserved Hepatocyte Recovery Medium (#CM7000, Thermo Fisher Scientific) and seeded on the coated UpCell dishes at a density of 6 × 10^4^ cells/cm^2^ and cultured overnight in an incubator at 37 °C with 5% CO2. PHHs were maintained in high-glucose DMEM (#10–017-CV, Corning) supplemented with 10% FBS (#10270-016, Gibco), 1% penicillin–streptomycin (#300-002-CI, Corning), 1.5% HEPES (#15630-080, Gibco), 0.2 µM glucagon (#G2044, Sigma-Aldrich, St. Louis, MO, USA), 0.1 μM dexamethasone (#D8893, Sigma-Aldrich), and 1% ITS premix (625 μg/mL insulin, 625 μg/mL human transferrin, 0.625 μg/mL selenous acid, 535 μg/mL linoleic acid, and 125 mg/mL bovine serum albumin; #354352, Corning) (PHH maintain medium). On the next day (day 3), HSCs (passage 2) were seeded at a density of 12 × 10^4^ cells/cm^2^ on UpCell dishes and were co-cultured overnight in an incubator at 37 °C and with 5% CO_2_.The medium was replaced daily until the day of sheet fabrication (day 0). On the day before sheet preparation (day 1), 6-well collagen I-coated microplates (#4810-010, IWAKI) were coated with iMatrix (0.25 μg/cm^2^) and incubated overnight at 37 °C. On the day of sheet fabrication (day 0), the microplates were washed twice with PBS at room temperature. UpCell dishes containing the co-culture were cooled at 20 °C for 30 min, following which the PHH/HSC sheets were detached from the surface. The detached sheets were transferred to the 6-well collagen I-coated microplates.

### Toxicity assay

We prepared four different types of media (A–D) to analyze the effects of glucose and free fatty acids (FFA) on hepatocytes. Normal glucose/insulin medium was composed of 1 g/L glucose and 6.25 ng/mL insulin (physiological concentration). High glucose/insulin medium was composed of 4.5 g/L glucose and 6.25 μg/mL insulin. FFA medium contained 300 μM oleic acid and 150 μM palmitic acid (Kohjin Bio, Saitama, Japan).

- A; Normal glucose/insulin/FFA (−) (Normal medium)
- B; High glucose/insulin/FFA (−) (Glucotoxicity medium)
- C; Normal glucose/insulin/FFA (+) (Lipotoxicity medium)
- D; High glucose/insulin/FFA (+) (Glucolipotoxicity medium)

Details of medium are described in the Supplementary Information.

PHH/HSC sheets were cultured in 1 mL of the indicated medium (A–D) in an incubator with 5% CO_2_ at 37 °C for 11 days. The medium was replaced daily.

### Simple co-culture

To confirm that the cell sheets maintained hepatocyte function, PHH/HSC simple co-culture was performed on the collagen dishes using glucolipotoxicity medium group as a control group. The seeding density was the same as that of cell sheets. Details regarding the co-culture are presented in the Supplementary Information.

### Morphological analysis

PHH/HSC sheets were imaged on day 11 using digital microscopy (VHX-900, KEYENCE Ltd., Osaka, Japan) and optical coherence tomography (IVS-2000, KEYENCE Ltd.). The area of the PHH/HSC sheets was analyzed using ImageJ ver. 1.53 (NIH, Bethesda, MD, USA). The sheet thickness of was calculated from the cross-sectional area by randomly imaging three different areas in each sheet using optical coherence tomography.

### Histological analysis

The PHH/HSC sheets were fixed with 4% paraformaldehyde (PFA), paraffin embedded, and cut into 5-μm thick slices. HE staining, E-cadherin/4′, 6-diamidino-2-phenylindole (DAPI) staining, Vimentin/DAPI staining, CK8/18 staining, alpha-smooth muscle actin (αSMA) staining, and p62 staining were performed on the fixed sections. Immunofluorescence staining was performed for E-Cadherin/DAPI and Vimentin/DAPI. Immunohistochemical staining using 3,3′-Diaminobenzidine-4HCI (DAB) was performed to detect CK8/18, αSMA, and p62. Formalin-fixed human liver (#HLFFPE-B, lot: HL170064) was purchased from Samsara Sciences (Solana Beach, CA, USA) and used as a positive control for p62 staining (Fig. S1). Details of the immunohistochemical analysis, antibodies, and dilutions are described in the Supplementary Information (Table S1). To evaluate hepatocyte ballooning, sections were stained with E-cadherin/DAPI and randomly imaged using a fluorescence microscope (BX51, Olympus, Tokyo, Japan). The cross-sectional area of hepatocytes with stained nuclei was measured using the ImageJ software. At least 15 hepatocytes were analyzed in each experiment under each condition. The average value was set at n = 1. The number of PHHs counted for each condition is described in the Supplementay Information (Table S2). To visualize accumulated lipids, oil red O staining was performed after fixing the sheets with 4% PFA. Briefly, oil red O powder (#154-02072, Wako) was dissolved in 60% isopropanol to prepare the staining solution. The staining solution was added onto PHH/HSC sheets and incubated for 20 min, following which the staining solution was washed off, and the sheets were observed under a phase-contrast microscope (ECLIPSE TS2, Nikon Ltd, Tokyo, Japan). To quantify the fat accumulation, oil red O stain was eluted from the sheets using 100% isopropanol and the absorbance was measured at 492 nm using a microplate reader. In addition, fat staining using the osmium method was performed for long-term storage. Oil red O staining and the osmium method are described in the Supplementary Information. For transmission electron microscopy (TEM), PHH/HSC sheets were fixed with 2% PFA and 2% glutaraldehyde in 0.1 M phosphate buffer. The samples were then washed and postfixed with 2% osmium tetroxide (0.1 M). Dehydration, infiltration, embedding, and polymerization were then performed. Samples were ultra-thin sectioned at 70 nm using an ultramicrotome (Ultracut UCT; Leica Vienna, Austria) and stained with 2% uranyl acetate followed by secondary staining with lead stain solution (#18-0875, Sigma-Aldrich). The samples were observed under TEM (JEM-1400Plus, JEOL Ltd., Tokyo, Japan).

### ELISA and assay

The bioactive substances present in the culture supernatant were analyzed using the TGF-β1 ELISA kit (#KE00002, Protein Tech, Rosemont, IL, USA), IL-8 ELISA kit (#KE00006, Protein Tech), and Sonic hedgehog (SHH) ELISA kit (#ab100639, Abcam) according to the manufacturer's protocol. The samples were analyzed on day 3 or day 4 when ballooning was first observed (Fig. S2). On day 11, PHH/HSC sheets were homogenized, and protein lysates were prepared. Lysates were analyzed using the p62 ELISA kit (#ADI-900-212, ENZO New York, NY, USA), and protein carbonyl ELISA kit (#STA-310, CELL BIOLABS, San Diego, CA, USA) according to the manufacturer's protocol. The BCA protein assay kit (#T9300A, Takara Bio) was used to determine the protein concentration in the lysates. The Human Albumin ELISA kit (#E88-129, Bethyl Laboratories, Inc., Waltham, MA, USA) and Urea assay kit (#DIUR-100, BioAssay Systems, Hayward, CA, USA) were used to assess hepatocyte function. The samples were analyzed on days 0, 1, 5, and 10.

### Real-time PCR

RNA was extracted from day 11 PHH/HSC sheets using the RNeasy Plus Mini Kit (#74134, QIAGEN, Venlo, Netherlands) and quantified using the NanoDrop One (Thermo Fisher Scientific). cDNA was prepared using the High-Capacity cDNA Reverse Transcription Kit (#4368814, Thermo Fisher Scientific) and ProFlex™ PCR System (Applied Biosystems). RT-PCR was performed using the ViiATM 7 Real-Time PCR System (Applied Biosystems) with glyceraldehyde-3-phosphate dehydrogenase (GAPDH: Hs02786624_g1), TNFα-induced protein 8 (TNFAIP8: Hs02621508_s1), and CYP1A2 (Hs00167927_m1). TaqMan assays (Thermo Fisher Scientific). GAPDH was used as internal normalizer.

### Statistical analyses

All experiments were performed at least thrice. The results were expressed as mean ± standard error of the mean (SEM). Intergroup differences between glucose and FFA exposed groups were analyzed using two-way ANOVA with Tukey’s post hoc test (for the comparison of more than two groups). All analyses were performed using the GraphPad Prism software (version 9.0, La Jolla, CA, USA). A *p* value < 0.05 was considered statistically significant.

## Results

### Effect of glucose and FFA on morphology of PHH/HSC sheets

**Figure 2:**
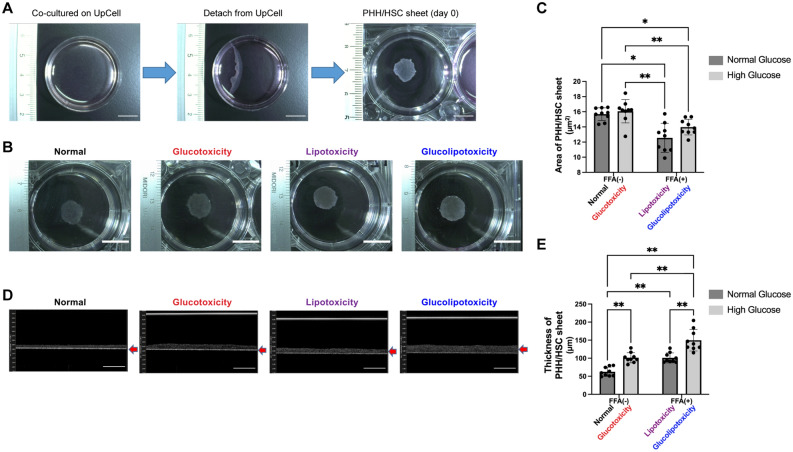
PHH/HSC sheet analysis. (**A**) PHH/HSC sheet fabrication. PHHs and HSCs were co-cultured on 35 mm or 24-well UpCell culture plates at 37 °C for 4 days. After, the UpCell plates were incubated at 20 °C for 30 min following which the PHH/HSC sheets were detached from the surface, contracted, and became 3D. Scale bar, 10 mm. (**B**) PHH/HSC sheet on day 11 fabricated on 35 mm UpCell dishes, viewed from the top. Scale bar, 10 mm. (**C**) Area of the PHH/HSC sheet fabricated on 24-well UpCell dishes. The area of sheets in the lipotoxicity and glucolipotoxicity groups was smaller than that in control and glucotoxicity groups, n = 9, 3 independent experiments, **p < 0.01, *p < 0.05. PHH: Primary human hepatocyte. (**D**) PHH/HSC sheet on day 11 fabricated on 24-well UpCell dishes, viewed from the side (OCT). Scale bar, 200 μm. (**E**) Thickness of the PHH/HSC sheet fabricated on 24-well UpCell dishes. The thickness was highest in the glucolipotoxicity group, n = 9, 3 independent experiments, **p < 0.01.

Following PHH and HSC co-culture, strong aggregation occurred, and cells were detached from the UpCell culture dishes, leading to the formation of PHH/HSC sheets (Fig. 2A). We tested the effects of glucose and FFA on the fabricated PHH/HSC sheets. In the non-FFA-treated group, the cells were more loosely connected to each other than in the FFA-treated group (Fig. 2B), whereas the area of PHH/HSC sheets was smaller in the FFA-treated-group (Fig. 2C) on day 11. There was a statistically significant difference in the FFA group (two-way ANOVA; FFA factor: F(1,8) = 55.19, *p* < 0.0001; glucose factor: F(1,8) = 3.494, *p* = 0.0985; FFA × glucose: F(1,8) = 1.106, *p* = 0.3237). The thickness of the PHH/HSC sheets was more in the glucolipotoxicity group than in other groups (Fig. 2D,E). There was a statistically significant difference in the glucose and FFA treatment groups [two-way ANOVA; FFA factor: F(1,8) = 85.67, *p* < 0.0001; glucose factor: F(1,8) = 59.97, *p* < 0.0001; FFA × glucose (4,8): F = 2.595, *p* = 0.1459]. These results indicate that FFA causes sheet aggregation and hyperglycemia along with FFA cause sheet thickening.

### Effects of glucotoxicity, lipotoxicity, and glucolipotoxicity on BH

**Figure 3:**
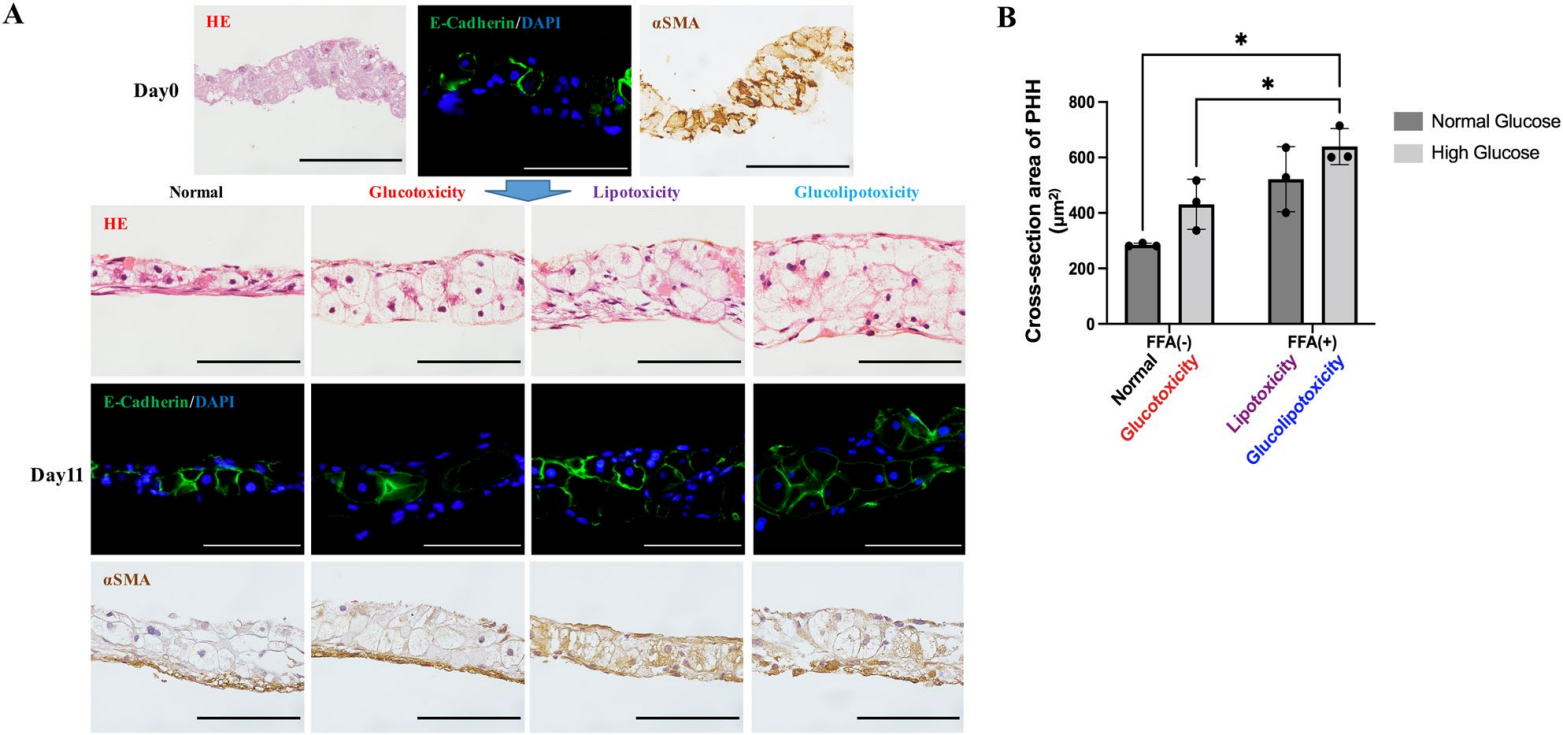
Histological analysis. (**A**) Representative images of HE staining and immunohistochemistry staining. Green: E-Cadherin, Blue: DAPI. Scale bar, 100 μm. HE: Hematoxylin–eosin, αSMA: Alpha-smooth muscle actin. (**B**) Evaluation of PHH ballooning. Ballooning was largest in the glucolipotoxicity group, n = 3, *p < 0.05. PHH: Primary human hepatocyte.

**Figure 4:**
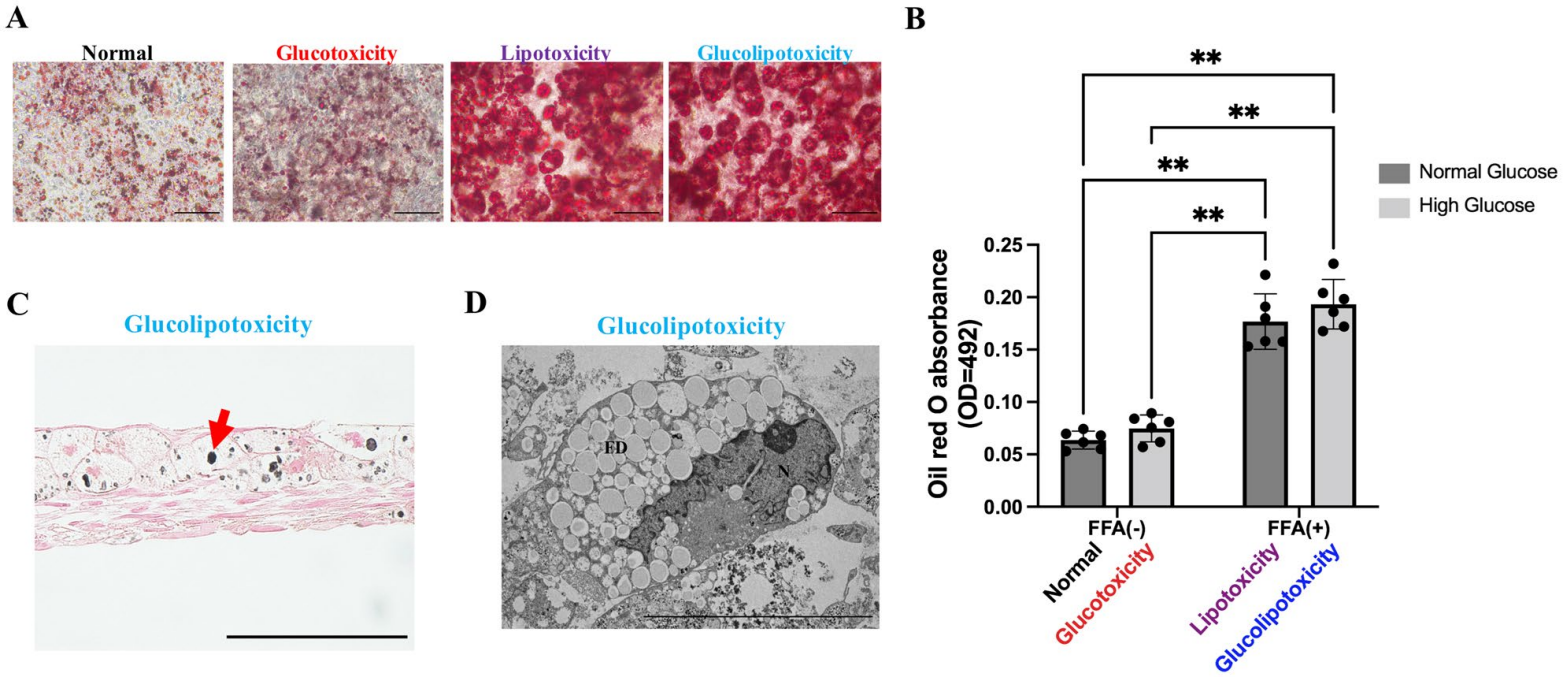
Evaluation of fat drop. (**A**) Representative images of oil red O staining. Scale bar, 200 μm. (**B**) Quantification of oil red O staining. Lipotoxicity and glucolipotoxicity groups showed significantly more oil red O accumulation than control and glucotoxicity groups, n = 6, 3 independent experiments, **p < 0.01. (**C**) Representative images of fat drops (Osmium method). Red arrow indicates fat drop. Scale bar, 100 μm. (**D**) Representative images of TEM. Fat accumulation was observed in the glucolipotoxicity group. Scale bar, 10 μm. N: nuclear, FD: fat drops.

HE staining showed cell layering and enlargement of PHHs with thinning of cytoplasm (ballooning) in the glucotoxicity, lipotoxicity, and glucolipotoxicity groups on day 11 (Fig. 3A). Vimentin/DAPI staining revealed Vimentin expression in the HSCs in each medium condition and was especially strong in Glucolipotoxicity (Fig. S3). Quantification of the cross-sectional area of PHHs was performed by E-Cadherin/DAPI staining. Results showed that PHHs were more than twofold enlarged in the glucolipotoxicity group than in the normal group (Fig. 3A,B). There was a statistical difference in the FFA group (two-way ANOVA; FFA factor: F(1,2) = 22.63, *p* = 0.0415; glucose factor: F(1,2) = 4.263, *p* = 0.1750; FFA × glucose: F(1,2) = 0.6088, *p* = 0.5169). αSMA staining labelled all cells, including periportal hepatocytes, in day 0 samples. At day 11, in the non-FFA-treated group, polarity in staining was observed as αSMA was localized to the basement membrane side, whereas in the FFA-treated group, staining was observed in all cells, including the periportal hepatocytes (Fig. 3A). Oil red O staining showed fat accumulation in the FFA-treated group (Fig. 4A,B). There was a statistically significant difference between the glucose and FFA treatment groups (two-way ANOVA; FFA factor: F(1,5) = 116.7, *p* = 0.0001; glucose factor: F(1,5) = 6.868, *p* = 0.0471; FFA × glucose: F(1,5) = 0.3450, *p* = 0.5825). Osmium method evaluation and electron microscopy revealed that fat droplets were not observed in HSCs, only in PHH (Fig. 4C,D). These results indicate that FFA induce the ballooning of PHHs, and hyperglycemia and FFA induce fat accumulation. Moreover, αSMA is strongly expressed during sheet detachment and FFA resolves polarity in αSMA localization.

### Visualization of MDBs

**Figure 5:**
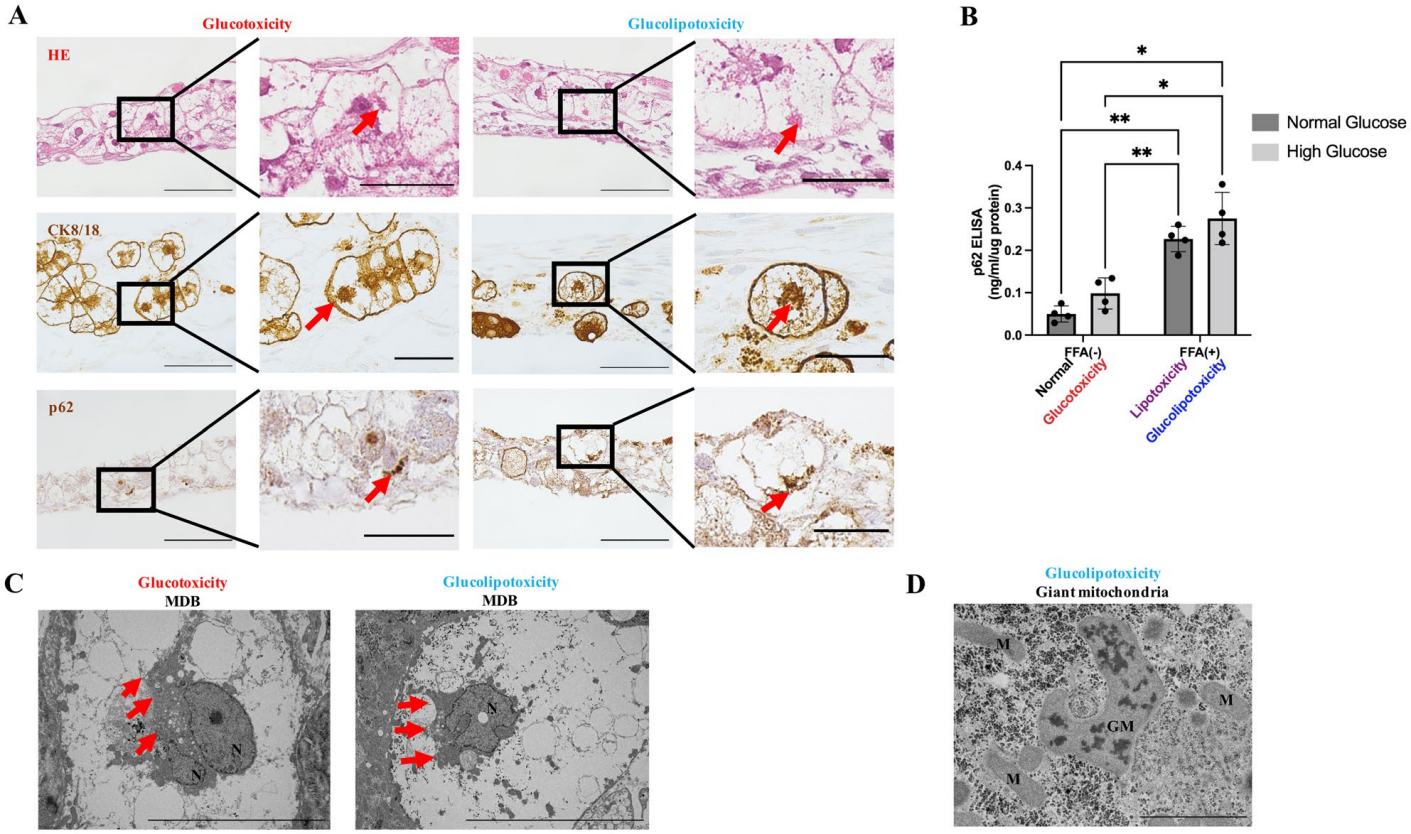
Identification of MDBs in vitro. (**A**) Representative images of HE staining and immunofluorescence staining. Eosinophilic bodies and CK8/18 aggregates were observed in glucotoxicity, lipotoxicity, and glucolipotoxicity groups. However, p62-positive bodies were identified only in the glucotoxicity and glucolipotoxicity groups. Red arrows indicate MDBs. Scale bar, 100 μm in normal views. Scale bar, 50 μm in enlarged views. HE: Hematoxylin–eosin, CK: Cytokeratin. (**B**) p62 protein quantification in PHH/HSC sheets by ELISA. p62 levels were significantly higher in the lipotoxicity and glucolipotoxicity groups compared to control and glucotoxicity groups. n = 4, 3 independent experiments, **p < 0.01, *p < 0.05. (**C**) Representative images of TEM. Red arrows indicate MDBs. MDBs were observed in the glucotoxicity and glucolipotoxicity groups. Scale bar, 10 μm. N: nuclear. (**D**) Representative images of TEM. Giant mitochondria was observed in the glucolipotoxicity group. Scale bar, 2 μm. M: mitochondria, GM: giant mitochondria with crystalline inclusion.

HE and CK8/18 staining showed eosinophilic bodies and CK8/18-positive aggregates in the BHs. p62 staining showed the presence of MDBs in the glucotoxicity and glucolipotoxicity groups (Fig. 5A). The expression level of p62 was markedly increased in the FFA-treated group (Fig. 5B). There was a statistically significant difference in the FFA and glucose treatment groups (two-way ANOVA; FFA factor: F(1,3) = 117.8, *p* = 0.0017; glucose factor: F(1,3) = 10.07, *p* = 0.0503; FFA × glucose: F(1,3) = 0.00915, *p* = 0.9963). TEM results showed MDBs as a dark-colored granular structure with radial distribution in the glucotoxicity and lipotoxicity groups (Fig. 5C). In addition, enlarged mitochondria with crystalline structures were observed in the glucolipotoxicity group (Fig. 5D).

### Functional analysis

**Figure 6:**
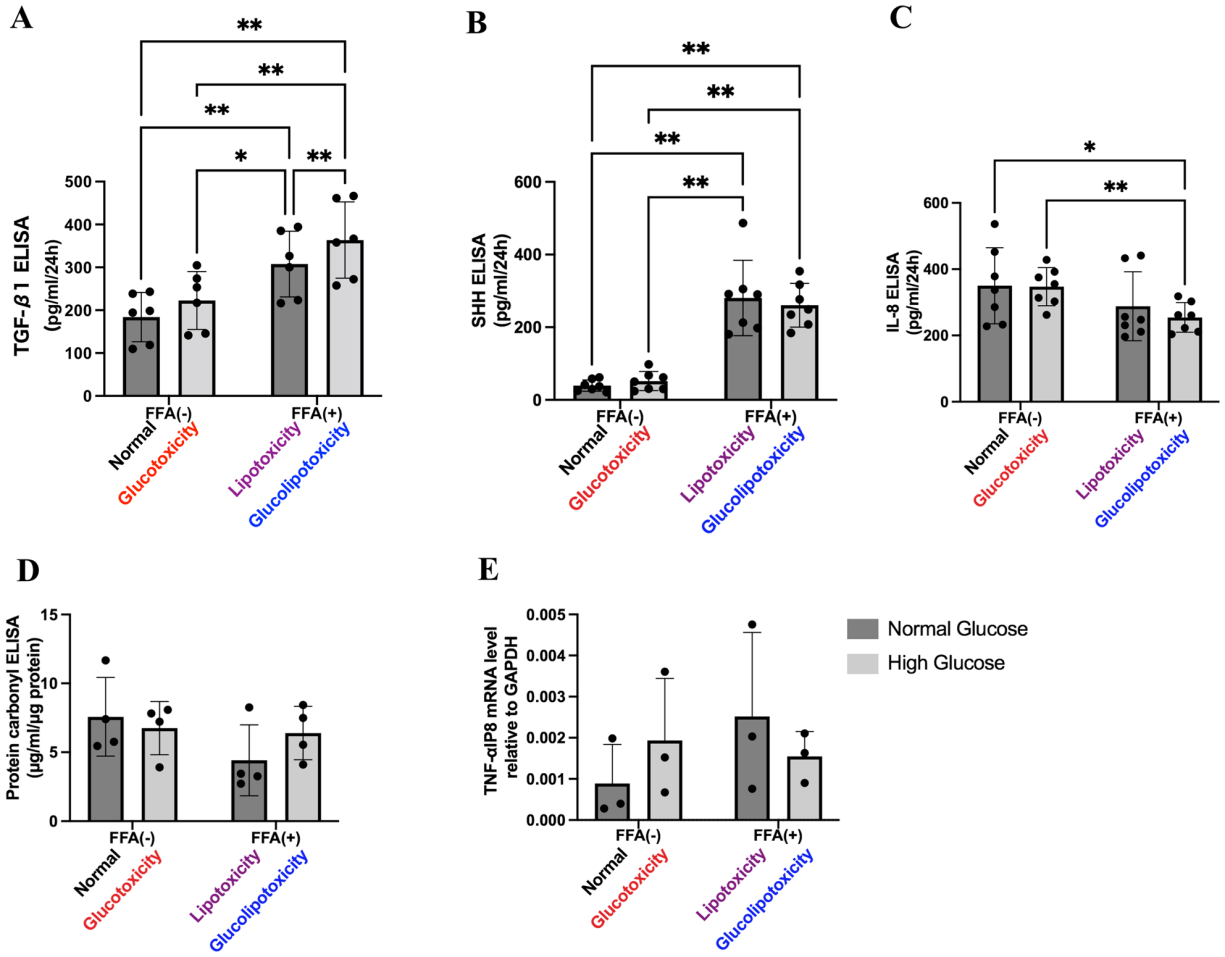
Functional analyses. (**A**) Evaluation of TGF-β1 levels in culture supernatant by ELISA. TGF-β1 levels were significantly higher in lipotoxicity and glucolipotoxicity groups compared to control and glucotoxicity groups. n = 6, 3 independent experiments, **p < 0.01, *p < 0.05. (**B**) Evaluation of SHH levels in culture supernatants by ELISA. SHH levels were significantly higher in lipotoxicity and glucolipotoxicity groups compared to control and glucotoxicity groups. n = 7, 3 independent experiments, **p < 0.01, *p < 0.05. SHH: Sonic hedgehog. (**C**) Evaluation of IL-8 levels in culture supernatant by ELISA. IL-8 levels were significantly higher in the lipotoxicity and glucolipotoxicity groups compared to control and glucotoxicity groups. n = 6, 3 independent experiments, **p < 0.01 *p < 0.05. (**D**) Evaluation of protein carbonyl levels in PHH/HSC sheet lysates by ELISA. Protein carbonyl levels were not significantly different between groups. n = 4. (**E**) Evaluation of TNFα-IP8 mRNA expression in PHH/HSC sheets by RT-qPCR. GAPDH was used as normalizer. No significant difference was observed between groups. n = 3, 3 independent experiments.

TGF-β1 secretion was the highest in the glucolipotoxicity group (Fig. 6A). There was a statistically significant difference in both glucose and FFA treatment groups (two-way ANOVA; FFA factor: F(1,5) = 73.66, *p* = 0.0004; glucose factor: F(1,5) = 34.93, *p* = 0.0020; FFA × glucose : F(1,5) = 1.121, *p* = 0.3382). SHH secretion was markedly elevated in the FFA-treated group (Fig. 6B). There was a statistically significant difference in both glucose and FFA treatment groups (two-way ANOVA; FFA factor: F(1,5) = 108.8, *p* < 0.0001; glucose factor: F(1,5) = 0.02621, *p* = 0.8767; FFA × glucose: F(1,5) = 0.6917, *p* = 0.4374). Moreover, IL-8 secretion was decreased in the FFA-treated group (Fig. 6C). There was a statistically significant difference in the FFA group (two-way ANOVA; FFA factor: F(1,6) = 19.68, *p* = 0.0044; glucose factor: F(1,6) = 0.4651, *p* = 0.5207; FFA × glucose: F(1,6) = 3.377, *p* = 0.1157). Protein carbonyl production and TNFα-IP8 expression levels were not significantly different between the groups (Fig. 6D,E). These results indicate that hyperglycemia and FFA induce the secretion of the fibrosis factor TGF-β1, and FFA induces SHH, but have no effect on inflammatory cytokines and oxidative stress.

### Hepatocyte function in the PHH/HSC sheets

Albumin secretion and urea synthesis were immediately deactivated in the simple co-culture, while they were maintained in cell sheets. The activity of CYP1A2 on day 11 was maintained compared to day 1 even in the presence of fatty acids. (Fig. S4).

## Discussion

The presence of a BH is an important finding in the histological diagnosis of human NASH. BHs are considered a special form of cellular degeneration that is caused by the accumulation of fatty acids and glycogen, abnormal protein accumulation due to unfolded protein response dysfunction, and autophagy suppression. However, the mechanism underlying BH formation is not well understood^5,14–17^. Histological features of BHs include hepatocyte ballooning (1.5- to twofold increase in size compared to normal hepatocytes), accumulation of fat droplets, abnormal arrangement of CK, abnormalities in organelles such as those in the mitochondria and endoplasmic reticulum, and the presence of MDBs^5,18–20^. In this study, we found that the exposure of PHH/HSC sheets to a glucolipotoxicity environment resulted in the production of BHs with features similar to those observed in human NASH. Functional analysis showed the involvement of fibrosis factors such as SHH and TGF-β1 but not the inflammatory cytokines such as TNFα and IL-8. Interestingly, BH was produced without the involvement of Kupffer cell (KC) or inflammatory cytokines. These results suggest that fibrosis is the major factor whereas inflammation is a supporting factor in BH formation. This observation is supported by the pathological correlation between fibrosis and BH formation^21,22^, and both correlate with clinical outcomes^10^.

Although fibrosis is the most important factor in BH formation, it is not well understood why exposure of PHH/HSC sheets to a glucolipotoxicity environment results in BH formation. In a previous study, it was shown that ballooning did not occur in co-culture sheets with 3T3-J2 cells^12^, which have been used as a feeder for maintaining PHH function^23^, but ballooning did occur in co-culture with NHDF, a fibroblast cell line^12^. These results suggest that fibrosis, hyperglycemia, and insulin resistance are important for ballooning. One of the factors that could produce BH was the change of co-culture cells to HSCs. HSCs are non-parenchymal cells of the liver and have a variety of functions^24^ One pathway of HSCs is activated by SHH, which functions as a protection against apoptosis when the liver is damaged, and are transformed into myofibroblasts^25^, to produce extracellular matrix (ECM) by increasing desmin and αSMA^24^. After ECM production, activated HSCs regress to inactive forms or undergo apoptosis^26^. However, HSCs continue to be activated under chronic damage such as fatty acid and oxidative stress and produce excessive ECM while further producing fibrosis-inducing factors such as TGF-β, causing liver fibrosis^24^. It has also been reported that hyperglycemia and insulin resistance indirectly promote fibrosis^27^. The action of HSCs as fibroblasts, chronic stimulation by fatty acids, and indirect effects of hyperglycemia and insulin resistance may have been factors involved in BH formation. Since ballooning does not occur in simple co-cultures^28,29^, it is assumed that there are some factors that lead to the production of BH in PHH/HSC sheets. We hypothesized that cells in the sheet will become three-dimensional (3D). This is supported by our observation that cells in the glucotoxicity, lipotoxicity, and glucolipotoxicity groups were layered compared to the normal group. Moreover, the 3D PHH/HSC sheets better represent the complex human liver due to enhanced cell-to-cell interaction^11,30,31^. Recently, the sinusoidal pressure hypothesis has been proposed, and a correlation between pressure and fibrosis has been shown^32^. We suspect that aggregation of PHH/HSC sheets during detachment exerts a strong force on cells (Fig. 2A), which in turn triggers fibrosis and HSC activation. In support of this, we observed that αSMA staining on day 0 of sheet fabrication showed strong activation of HSCs (Fig. 3A). During PHH/HSC sheet fabrication in the absence of FFA, αSMA was mainly localized at the basal side, while in the presence of FFA, αSMA showed no polarity and was localized to all regions including the periphery of hepatocytes (Fig. 3A). Thus, during sheet fabrication, stress due to external forces and glucolipotoxicity environment may have contributed to BHs formation.

MDBs are not specific to NASH but are also observed in various other diseases such as alcoholic steatohepatitis, liver cell carcinoma, and Wilson’s disease^33^. MDBs are thought to be formed as a result of the complex interaction between several factors, such as fibrosis, inflammation, suppression of autophagy, and cellular stress. All these factors lead to an imbalance in protein homeostasis within the cell and accumulation of abnormal intracellular proteins such as ubiquitin and p62 in addition to CK^33–35^. However, the detailed underlying mechanisms remain unclear. Autophagy, a mechanism through which homeostasis is maintained by the degradation of unwanted cytoplasmic components and abnormal proteins, is thought to be important for MDB formation^36^. In general, damaged organelles and abnormal proteins are modified with p62 and degraded. Once autophagy is inhibited, degradation cannot be performed, and p62 accumulates in the cell, which leads to MDB formation^37^. Histological and functional analyses in our study showed that Ballooning, fibrosis, and fat accumulation are mainly induced by FFA exposure. The presence of MDBs suggest the involvement of hyperglycemia and hyperinsulinemia because these bodies appeared in the glucotoxicity and glucolipotoxicity groups. Interestingly, MDBs appeared in the glucotoxicity group, even though autophagy was only mildly suppressed as evidenced by a slight increase in p62 expression. It might be possible that mechanisms involved in autophagy suppression are different in PHHs and PHH/HSC sheets. It has been reported that autophagy is suppressed in hepatocytes with fat accumulation in human NASH^38^, which is consistent with the results of this study. We investigated the presence of MDBs in PHHs from three different donors and found that MDBs were present in PHHs from only one donor (HU8317), even though all donors showed BH phenotypes such as ballooning and fatty degeneration. Clinically, MDBs have been reported only in 10%–70% of patients with NASH^33,39^. Reportedly, MDBs are particularly difficult to observe in pediatric NASH^40^ and in cases of acute liver injuries such as acute hepatitis and drug poisoning^33^. This suggests that aging-related cumulative oxidative injury or genetic variables may play a contributing role. The background of the PHH lot used in this experiment was reviewed (Table S3), but no clear factor causing donor differences was found. On the other hand, in vitro, p62 aggregation by NF-κB activation^41^ and dysfunction of the ubiquitin–proteasome system are relevant to MDB^33^. Therefore, there may be differences in the expression of ubiquitin and NF-κB among lots.

Although various molecules are involved in NASH development and progression, the key factors are thought to be metabolic abnormalities due to hepatocyte steatosis, progression of fibrosis due to HSC activation, and inflammatory stimulation by KCs^2^. This study has some limitations. Our PHH/HSC model Our PHH/HSC model only partially represented NASH; i.e., it only demonstrated BHs. It could partially reproduce hepatocyte steatosis and fibrosis but could not reproduce inflammation. Since no inflammation was observed in our in vitro model, oxidative stress and endoplasmic reticulum stress could not be detected as well. Further, reportedly, KCs and inflammatory cytokines can cause fibrosis and cellular damage^42,43^. Therefore, a tri-culture model with KCs is essential to better reproduce human NASH, and further research is required to evaluate cell behavior in a tri-culture model. Our model could produce BHs and fibrosis in approximately 2 weeks; therefore, it could also be used as a fibrosis model with BHs^13^. Furthermore, patients with MDBs have been reported to have a worse prognosis, and thus, the presence of MDBs can be a clinically important indicator^44^. Hence, the use of healthy hepatocytes from the donors in this model and screening for the appearance of MDBs may be applied to predict the prognosis of the donor after future development of NASH.

In summary, in vitro BH can be produced by exposing PHH/HSC sheets to a glucolipotoxicity environment. BH formation in this model is not due to inflammatory cytokines production but due to transformation of cells to a 3D structure and fibrosis. Our model will further help in understanding the mechanisms underlying BH formation. In the future, this model could be further developed by incorporating inflammatory cells in the PHH/HSC sheets and creating a new type of in vitro NASH model with BHs.

## Supplementary Information

Supplementary Information.

## Data Availability

Data supporting the findings of this study are available in the article and Supplementary Information files, or from the corresponding author upon request.

## Abbreviations

NAFLD: Nonalcohoic fatty liver disease
NASH: Nonalcoholic steatohepatitis
BH: Ballooned hepatocyte
HE: Hematoxylin and eosin
CK: Cytokeratin
MDB: Mallory–Denk body
PHH: Primary human hepatocyte
NHDF: Normal human dermal fibroblast
HSC: Hepatic stellate cell
FFA: Free fat acid
PFA: Paraformaldehyde
αSMA: Alpha smooth muscle actin
DAB: 3,3′-Diaminobenzidine-4HCI
TEM: Transmission electron microscopy
SHH: Sonic Hedgehog
KC: Kupffer cell
ECM: Extracellular matrix
