# Supplementary material for: In vitro ballooned hepatocytes can be produced by primary human hepatocytes and hepatic stellate cell sheets
Source: Sci Rep. 2022 Mar 29;12:5341. doi: 10.1038/s41598-022-09428-x (PMC8964766; doi:10.1038/s41598-022-09428-x)
Supplement: Supplementary file 1 — Supplementary Information. [file 41598_2022_9428_MOESM1_ESM.docx]

**Supplementary Information**

***In vitro* ballooned hepatocytes can be produced by primary human hepatocytes and hepatic stellate cell sheets**

**Authors**

Nobuhiro Hasui^1,2^: nobuhiro-hasui@ks.kyorin-u.ac.jp

＊Katsuhisa Sakaguchi^3^: katsuhisa@toki.waseda.jp

Tetsuya Ogawa^4^: Tetsuya_Ogawa@mb1.nkc.co.jp

Yoshihiro Sakamoto^5^: yosakamo@ks.kyorin-u.ac.jp

＊Tatsuya Shimizu^1^: shimizu.tatsuya@twmu.ac.jp

**Affiliations**

^1^Institute of Advanced Biomedical Engineering and Science, TWIns, Tokyo Women's Medical University, Tokyo, Japan.

^2^Department of Gastrointestinal and General Surgery, Kyorin University School of Medicine, Tokyo, Japan.

^3^Department of Integrative Bioscience and Biomedical Engineering, Graduate School of Advanced Science and Engineering, TWIns, Waseda University, Tokyo, Japan.

^4^Ogino Memorial Laboratory, Nihon Kohden Co., Ltd., Tokyo, Japan.

^5^Department of Hepato-Biliary-Pancreatic Surgery, Kyorin University Hospital, Tokyo, Japan.

**Corresponding author**

Katsuhisa Sakaguchi

Email: katsuhisa@toki.waseda.jp

Tatsuya Shimizu

**Keywords:** Nonalcoholic steatohepatitis, Ballooned hepatocyte, *in vitro* model, glucolipotoxicity, co-culture, Mallory-Denk body

**Supplementary Methods**

**Cell-sheet medium**

The STLB solution (ITS premix without insulin) was prepared by dissolving selenous acid (0.625 μg/mL; #211176, Sigma-Aldrich), transferrin (625 μg/mL; #T8158, Sigma-Aldrich), linoleic acid (535 μg/mL #L1012, Sigma-Aldrich), and bovine serum albumin (125 mg/ml; #A8806, Sigma-Aldrich).

A; Normal glucose/insulin/FFA (-) (Normal medium): Low-glucose DMEM (#11885076, Gibco) with 5% FBS, 1% penicillin-streptomycin, 0.2 µM glucagon, 0.1 µM dexamethasone, 1% STLB solution, and 0.1% glucotoxicity medium.

B; High glucose/insulin/FFA (-) (Glucotoxicity medium): High-glucose DMEM with 5% FBS, 1% penicillin-streptomycin, 0.2 µM glucagon, 0.1 µM dexamethasone, and 1% ITS premix.

C; Normal glucose/insulin/FFA (+) (Lipotoxicity medium): FFA dissolved low-glucose DMEM with 5% FBS, 1% penicillin/streptomycin, 0.2 μM glucagon, 0.1 μM dexamethasone, 1% STLB solution, and 0.1% glucolipotoxicity medium.

D; High glucose/insulin/FFA (+) (Glucolipotoxicity medium): FFA-dissolved high-glucose DMEM with 5% FBS, 1% penicillin/streptomycin, 0.2 µM glucagon, 0.1 µM dexamethasone, and 1% ITS premix.

**Simple co-culture**

On the day prior to primary human hepatocytes (PHH) seeding, collagen I-coated dishes were coated with iMatrix (0.25 μg/cm^2^) and incubated overnight at 37 °C. The coated dishes were washed twice with phosphate-buffered saline (PBS) at room temperature. The PHHs were seeded on the coated dishes at a density of 6 × 10^4^ cells/cm^2^ and cultured overnight in an incubator at 37 °C with 5% CO_2_ (day 0), and maintained PHH maintainance medium. On the next day (day 1), HSCs (passage 2) were seeded at a density of 12 × 10^4^ cells/cm^2^ on dishes and were co-cultured overnight in an incubator at 37 °C and with 5% CO_2_. From the next day(day 2), the co-culture was maintained using the glucolipotoxicity medium. The medium was changed daily and the culture was maintained for 10 days. Co-culture samples were transferred to gelatin gel, and fixed.

**E-cadherin/DAPI staining**

The 5-μm thick sections were deparaffinized and treated with HistoVT One (#06380-05, Nakalai Tesque, Kyoto, Japan) for antigen activation. After blocking with Blocking One Histo (#06349-64, Nakalai Tesque) for 2 h, sections were incubated overnight with anti-E-cadherin antibody. Following this, the sections were incubated with Alexa Fluor 488-conjugated goat anti-rabbit IgG (H+L) highly cross-adsorbed secondary antibody for 2 h. Sections were mounted using the ProLong™ Diamond Antifade Mountant with DAPI (#P36962, Invitrogen).

**Vimentin/DAPI staining**

The 5-μm thick sections were deparaffinized and treated with HistoVT One for antigen activation. After blocking with Blocking One Histo for 2 h, sections were incubated overnight with anti-Vimentin antibody. Following this, the sections were incubated with Alexa Fluor 488-conjugated goat anti-rabbit IgG (H+L) highly cross-adsorbed secondary antibody for 2 h. Sections were mounted using the ProLong™ Diamond Antifade Mountant with DAPI.

**CK8/18 staining**

The 5-μm thick sections were deparaffinized, antigen-activated, and treated with 3% hydrogen peroxide for 15 min to remove endogenous enzymes. Blocking was performed using Blocking I (#03953-95, Nakalai Tesque) for 15 min. Sections were incubated with NCL-L-AE1/AE3-601 primary antibody overnight followed by incubation with Histofine simple stain MAX-PO secondary antibody for 30 min. DAB was used for color development, and Mayer’s hematoxylin was used for nuclear staining.

**αSMA staining**

The 5-μm thick sections were deparaffinized and treated with 3% hydrogen peroxide to remove endogenous enzymes. Blocking was performed using Blocking I for 15 min. Sections were then incubated with mouse anti-human smooth muscle actin monoclonal antibody (clone1A4) overnight. Following this, sections were incubated with Histofine simple stain MAX-PO secondary antibody for 30 min. DAB was used for color development, and Mayer’s hematoxylin was used for nuclear staining.

**p62 staining**

The 5-μm thick sections were deparaffinized and treated with 3% hydrogen peroxide to remove endogenous enzymes. Blocking was performed using Blocking I for 15 min. Sections were incubated with rabbit anti-p62 polyclonal antibody, overnight followed by incubation with Histofine simple stain MAX-PO(R) secondary antibody for 30 min. DAB was used for color development, and Mayer’s hematoxylin was used for nuclear staining.

All antibodies used are listed in Table S1.

**Oil red O staining**

Oil Red O powder (#154-02072, Wako) was dissolved in 60% isopropanol (3 mg/ml) and filtered to prepare the staining solution. The fixed PHH/HSC sheets were washed with PBS and incubated with the staining solution for 20 min with shaking. The staining solution was removed, and the PHH/HSC sheets were washed with distilled water and 60% isopropanol and imaged. To quantify oil red O staining, isopropanol (100%) was added to the stained sheets and incubated for 5 min to elute the stain. The absorbance of the colored isopropanol was measured using a microplate reader. Absolute isopropanol solution was used as the blank.

**Osmium method**

PHH/HSC sheets were fixed with 2% paraformaldehyde and 2% glutaraldehyde in 0.1 M phosphate buffer. The samples were then washed and postfixed with 2% osmium tetroxide (0.1 M) and 5% potassium dichromate, embedding in paraffin, and cut into 5-μm thick slices. The 5-μm thick sections were deparaffinized and treated with 5% periodic acid. Mayer’s hematoxylin and Eosin were used for contrast staining.

**Supplementary Tables**

**Table S1.** Antibodies used in this study

| **Antibody** | **Target** | **Supplier** | **Reference** | **RRID** | **Dilution** |
| --- | --- | --- | --- | --- | --- |
| Primary antibodies | E-Cadherin | Abcam | Ab40772 | AB_731493 | 1:100 |
|  | Vimentin | Abcam | ab92547 | AB_10562134 | 1:100 |
|  | AE1/AE3 | Leica | NCL-L-AE1/AE3-601 | N/A | 1:500 |
|  | αSMA | DAKO | M0851 | AB_2223500 | 1:500 |
|  | P62 | MBL | PM045 | AB_1279301 | 1:20000 |
| Secondary antibodies | Anti-E-Cadherin antibody | Thermo Fisher Scientific | A-11034 | AB_2576217 | 1:200 |
|  | Anti-AE1/AE3 antibody | NICHIREI | 414171F | AB_2811178 | undiluted |
|  | Anti-αSMA antibody | NICHIREI | 414171F | AB_2811178 | undiluted |
|  | Anti-P62 antibody | NICHIREI | 424144 | AB_2868561 | undiluted |

**Table S2.** Number of counted BHs

| **Round** | **Normal**  **group** | **Glucotoxicity**  **group** | **Lipotoxicity**  **group** | **Glucolipotoxicity group** |
| --- | --- | --- | --- | --- |
| **1st** | 19 | 18 | 27 | 23 |
| **2nd** | 19 | 21 | 38 | 43 |
| **3rd** | 39 | 45 | 28 | 26 |

**Table S3.** Clinical background of the PHH lot

| **Lot** | **Gender**  **Year**  **Race** | **BMI** | **Smoke**  **Alcohol**  **Drag** | **Medication** | **Serology** | **Cause of death** |
| --- | --- | --- | --- | --- | --- | --- |
| **HU8200** | Male  52  African-American | 17 | (+)  (+)  (+) | (-) | CMV | Anoxia |
| **HU1652** | Male  70  Caucasian | 30 | (+)  (+)  (-) | Allopurinol  Sertraline  Vitamin C  Fish oil capsule | (-) | N/A |
| **HU8317** | Male  59  Caucasian | 21.4 | (+)  (-)  (-) | (-) | (-) | SAH |

BMI: Body Mass Index, CMV: cytomegalovirus, SAH: subarachnoid hemorrhage


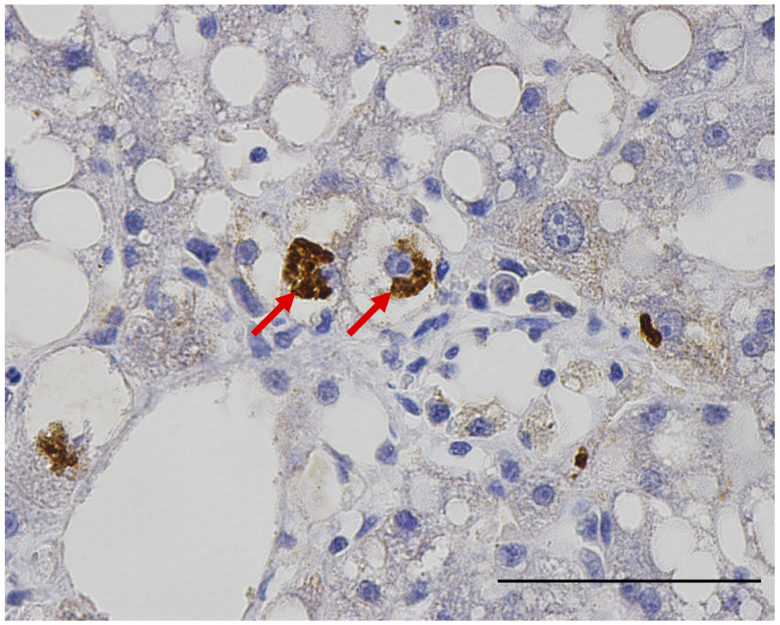


**Supplemental Figure 1.** Positive control of p62 staining

Red arrows indicate MDB. Scale bar, 100 μm.


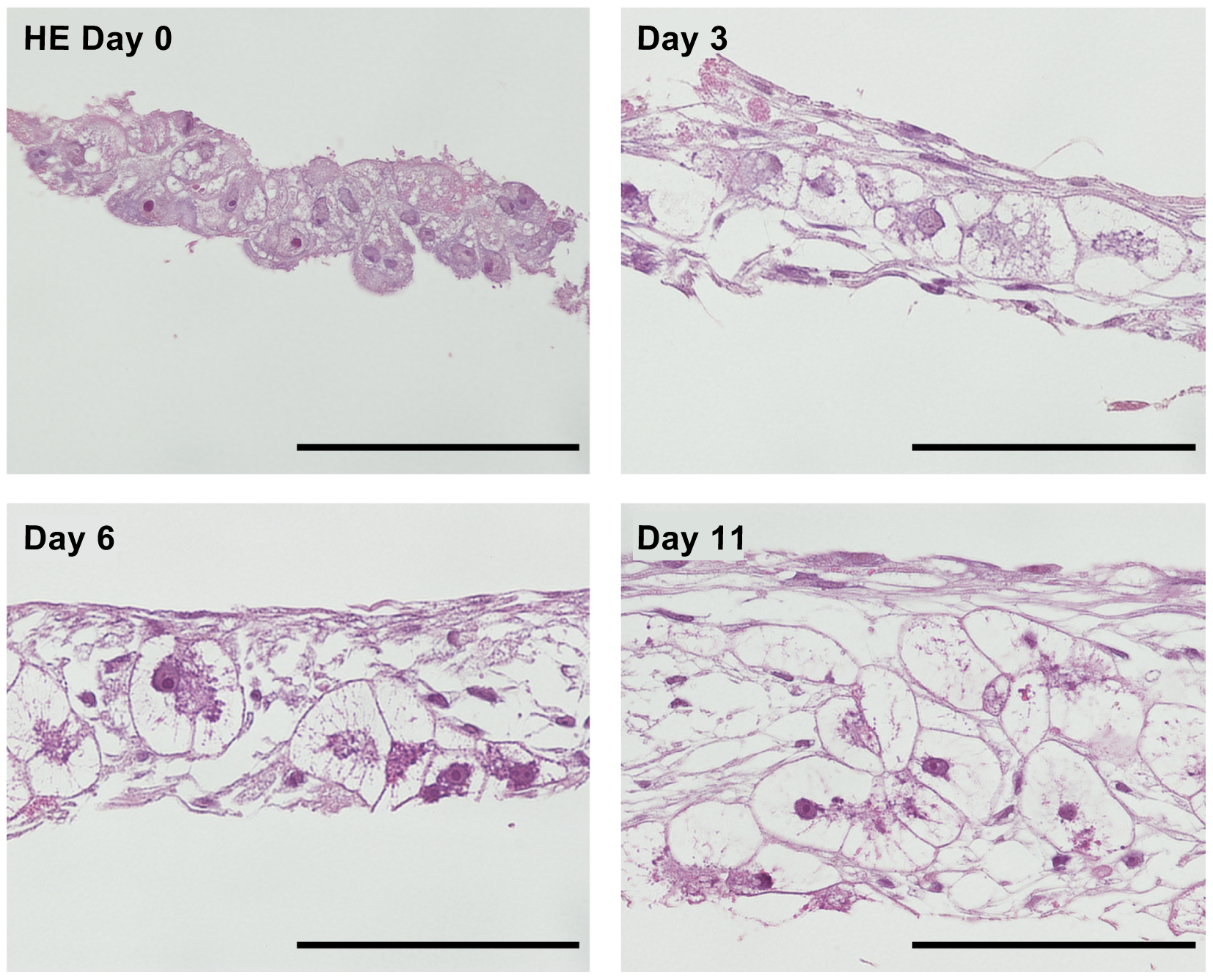


**Supplemental Figure 2.** Ballooning changes over time (glucolipotoxicity).

Ballooning occurred on day 3, and was even larger on days 6 and day 11. Scale bar, 100 μm.

**Supplemental Figure 3.** Vimentin/DAPI staining (day 11)

Green: Vimentin, Blue: DAPI. Scale bar, 100 μm.

**Supplemental Figure 4.** Hepatocyte function in the PHH/HSC sheet (glucolipotoxicity)

(A) Urea synthesis in PHH/HSC co-culture and PHH/HSC sheets. PHH/HSC co-culture: n=3, 2 independent experiments. PHH/HSC sheets: n=6, 3 independent experiments.

(B) Albumin secretion in PHH/HSC co-culture and PHH/HSC sheets. PHH/HSC co-culture: n=3, 2 independent experiments. PHH/HSC sheets: n=6, 3 independent experiments.

(C) Evaluation of CYP1A2 mRNA expression in PHH/HSC sheets by RT- qPCR. GAPDH was used as normalizer. day 1: n=2, 2 independent experiments. day 11: n=3, 3 independent experiments.
